# Effect of *Wolbachia* Infection and Adult Food on the Sexual Signaling of Males of the Mediterranean Fruit Fly *Ceratitis capitata*

**DOI:** 10.3390/insects13080737

**Published:** 2022-08-17

**Authors:** Georgios A. Kyritsis, Panagiota Koskinioti, Kostas Bourtzis, Nikos T. Papadopoulos

**Affiliations:** 1 Laboratory of Entomology and Agricultural Zoology, Department of Agriculture Crop Production and Rural Environment, University of Thessaly, Phytokou St., 38446 New Ionia, Greece; 2 Insect Pest Control Laboratory, Joint FAO/IAEA Centre of Nuclear Techniques in Food and Agriculture, 2444 Seibersdorf, Austria

**Keywords:** medfly, endosymbiotic bacteria, pheromone, sexual behavior, Incompatible Insect Technique, Sterile Insect Technique

## Abstract

**Simple Summary:**

The Mediterranean fruit fly (medfly) can infest many plant species and survive in different climatic conditions, thus leading to its invasion in new geographical regions and causing great economic losses in fruit production. Insecticides have been the major approach for the suppression of medfly populations for decades. However, concerns regarding insecticide impact on the environment led to the development of environmentally friendly techniques that rely on field releases of sterile males that produce no viable offspring when mated with wild females. One method of male sterilization is their infection with bacteria such as *Wolbachia* that induce sterility in infected males when crossed with uninfected wild females. The released sterile males should be competitive with wild males for mating with wild females. Therefore, it is important to evaluate the effect of *Wolbachia* on traits that determine male mating success, such as sexual pheromone signaling, and assess potential ways to improve mating success (i.e., enrichment of male diet with protein). In this study, we demonstrate that *Wolbachia* infection decreases sexual signaling frequency, but protein-enriched diets cannot counteract this negative effect. Our results contribute to the assessment of *Wolbachia* infection as an additional tool for population suppression of insect pests.

**Abstract:**

Sexual signaling is a fundamental component of sexual behavior of *Ceratitis capitata* that highly determines males’ mating success. Nutritional status and age are dominant factors known to affect males’ signaling performance and define the female decision to accept a male as a sexual partner. *Wolbachia pipientis*, a widespread endosymbiotic bacterium of insects and other arthropods, exerts several biological effects on its hosts. However, the effects of *Wolbachia* infection on the sexual behavior of medfly and the interaction between *Wolbachia* infection and adult food remain unexplored. This study was conducted to determine the effects of *Wolbachia* on sexual signaling of protein-fed and protein-deprived males. Our findings demonstrate that: (a) *Wolbachia* infection reduced male sexual signaling rates in both food regimes; (b) the negative effect of *Wolbachia* infection was more pronounced on protein-fed than protein-deprived males, and it was higher at younger ages, indicating that the bacterium regulates male sexual maturity; (c) *Wolbachia* infection alters the daily pattern of sexual signaling; and (d) protein deprivation bears significant descent on sexual signaling frequency of the uninfected males, whereas no difference was observed for the *Wolbachia*-infected males. The impact of our findings on the implementation of Incompatible Insect Technique (IIT) or the combined SIT/IIT towards controlling insect pests is discussed.

## 1. Introduction

The Mediterranean fruit fly (medfly), *Ceratitis capitata* (Wiedemann), is a highly polyphagous species, known to infest more than 350 plant species and causes severe damage to a wide variety of fruits and vegetables worldwide [1,2,3]. Its ability to use different hosts and adapt in a wide range of climatic conditions gives the medfly a high invasion potential and allows its survival in both tropical and temperate climates [4,5,6]. Due to its global distribution and the great economic impact on fruit production and trade, the medfly has become the most studied member of the true fruit flies (Tephritidae family) and a major target species of multiple environmentally friendly population suppression strategies. The Sterile Insect Technique (SIT) is one of the most successful tools of such strategies, and the medfly has been the model species for the development and implementation of SIT in Tephritidae Integrated Pest Management (IPM) programs [7].These approaches are based on mass production, sterilization, and release of sterile males in the field where they compete with wild males for mating with wild females. Crosses between the released sterile males and the wild females are infertile, leading to the decline of the natural population in the target area [8]. Traditionally, sterilization of males in SIT applications is achieved by irradiation-induced chromosomal breaks that cause dominant lethal mutations in the sperm of the released males [9,10].

The success of population suppression methods depends heavily on the biological quality, sexual behavior, and the overall capacity of the released sterile males to perform successful courtships in the field [11]. *Ceratitis capitata* is a species with complex courtship behavior and its mating depends to a great extent on male sexual performance [12,13]. Many studies have demonstrated that the processes of mass production and sterilization of the released males may affect their sexual performance and competitiveness against the wild males [14,15,16,17,18]. For instance, irradiation of males may reduce male mating competitiveness [17,19,20,21] and female receptivity of irradiated males, and may increase female remating propensity [22] in medfly. Alternative technologies such as the release of insects carrying a dominant lethal (RIDL) were developed in an effort to avoid the negative effect of irradiation on male sexual performance [23]. However, it has been shown that some RIDL and other transgenic strains demonstrated similar male mating competitiveness with traditional SIT strains under field–cage conditions [24,25]. Therefore, it is crucial to investigate the efficiency of each strain case by case to reach more safe conclusions regarding their efficiency [26]. Additionally, there are still issues related to regulatory restrictions and public acceptance of genetically modified insect technology worldwide [27].

A fundamental component of the medfly’s sexual behavior and courtship performance is male sexual signaling. Males aggregate on the host plant leaves and form “leks” where they display their presence through complex pheromone emissions (known as sexual signaling or calling) to attract females seeking mating opportunities [28,29]. In this competitive surrounding, females actively select the mate that corresponds to their evolutionary-determined demands. Previous studies have demonstrated that only a small proportion of males account for the majority of all matings within a lek [30], while increased sexual calling propensity is positively correlated with high male mating success [30,31,32]. This evidence indicates the critical effect of male sexual signaling on the female decision to accept a male as a sexual partner.

Complex reproductive displays such as sexual signaling can be expensive for male insects [33,34,35]. The production of the molecular and biochemical machinery that contributes to male pheromone synthesis requires the extended use of nutrient reserves such as amino acids and might be affected by the nutritional status of males [36]. The determinant effect of adult diet on male sexual performance has been demonstrated for many Tephritid species such as *Anastrepha* [37,38,39,40,41], *Bactrocera* [42,43,44], and *Rhagoletis* [45,46]. For example, high protein content in a male adult diet increases pheromone production, accelerates the onset of pheromone calling and reproductive development in *Anastrepha* spp. [40,47], and enhances sexual performance in *Bactrocera* spp. [42,48,49]. Several studies of the Mediterranean fruit fly have shown that adult protein-rich diets enhance male sexual performance attributes such as sexual signaling [50,51,52], participation in leks, mating success and duration, and inhibition of female remating [34,53,54,55,56,57,58]. In addition to the effect of adult diets, there are also studies that demonstrate the determinant effect of larval diet content on both male sexual performance [59] and other insect fitness traits [60,61], and the nutritional adaptation that occurs during long-term laboratory rearing that leads to significant variation across different fitness-related traits [59,61]. Another critical factor that affects sexual calling behavior is the age of the males. Studies in *C. capitata* indicate that younger males display higher calling activity [62] and are preferred over older males by young females [63]. Therefore, it is crucial to consider both the nutritional status and the age of the released sterile males for the assessment of their mating success in any population control approach.

In addition to the widely used SIT, some insect population control approaches utilize the Incompatible Insect Technique (IIT) that is based on the infection of the insect reproductive tissues with the Alphaproteobacteria *Wolbachia pipientis*. *Wolbachia* are maternally inherited endosymbiotic bacteria that can infect insects, isopods, spiders, and filarial nematodes [64,65,66,67]. The most pronounced *Wolbachia* effect on their hosts is the ability to manipulate host reproduction using several strategies, such as parthenogenesis [68,69], feminization [70,71], male killing [72,73,74,75], and cytoplasmic incompatibility (CI), to promote its transmission and increase its occurrence in the population [64,76,77]. CI is the most common reproductive phenotype induced by *Wolbachia* in fruit flies and is expressed as embryonic mortality occurring in crosses of *Wolbachia*-infected males with either uninfected females (unidirectional CI) or females infected with a different (incompatible) *Wolbachia* strain (bidirectional CI) [78,79]. IIT approaches rely on the CI effect and the release of *Wolbachia*-infected males that are unable to produce viable offspring when mated with uninfected wild females, thus leading to population decrease [80].

The ability of *Wolbachia* to infect several other somatic tissues, such as salivary glands, gut, fat body, hemocytes, Malpighian tubules, and especially the nervous system, is an indication that the endosymbiont can induce additional changes in the host’s behavior and fitness [81,82]. These changes could affect (positively or negatively) the outcome of IIT approaches. Various studies have shown that *Wolbachia* alters traits associated with sleep [83,84,85], feeding [86], locomotory behavior [87,88], learning and memory capacity [89,90], protection of its host against other pathogens [91,92,93,94,95,96,97], response to olfactory cues [98], and mating preference and behavior [99,100,101,102,103]. For instance, studies in *Drosophila* demonstrated that *Wolbachia*-infected males show increased mating rates and competitiveness compared to uninfected males [104,105]. Furthermore, infected *D. paulistorum* females show strong mating preference to males infected with the same *Wolbachia* variant [101], but this preference is lost with the decrease of *Wolbachia* titer in males [106]. Similar results of pre-mating assortative mating have been demonstrated by Koukou et al. (2006) in *D. melanogaster* [100]. Post-mating alterations were also observed in *D. melanogaster* females that showed reduced remating receptivity, when previously mated with *Wolbachia*-infected males [107,108]. Such assortative mating behaviors in insects could be explained by differences in the pheromone profiles of males or females [109,110,111] and *Wolbachia* has indeed been associated with such differences in the pheromone profile of *D. paulistorum* males [106,112] and *Armadillidium vulgare* females [113,114].

The idea of implementing *Wolbachia* in insect pest control strategies was first introduced in the 1970s [78,115]. Since there is no evidence of infection in wild *C. capitata* populations [116] (but see [117]), *Wolbachia*-infected males were proposed to be used for IIT or combined SIT/IIT applications [118,119]. Artificially *Wolbachia*-infected medfly lines were established via the microinjection of cytoplasm originating from naturally infected *R. cerasi* embryos (donor) in medfly eggs that came from (a) a non-infected laboratory strain [118] and (b) a non-infected Genetic Sexing Strain (GSS) used in medfly SIT [80]. The established lines could induce 100% cytoplasmic incompatibility in proper crosses [80,118]. The influence of *Wolbachia* artificial infection in *C. capitata* fitness and biological quality has been addressed by a few studies. Decreased egg-to-larva and egg-to-adult survival rates, shortened egg-to-adult developmental time, reduced adult lifespan and female fecundity, and altered male mating competitiveness and flight ability were among the effects of *Wolbachia* infection [120,121,122]. Some of the alterations in these traits differed depending on both the strain of *Wolbachia* that was introduced in the medfly lines and the genetic background of these lines [122]. Moreover, a recent study by Dionysopoulou et al. demonstrated that *Wolbachia* decreases immature survival and increases immature developmental time when immature development takes place on natural host fruits instead of artificial diets [123]. Since any sterility-induced method (SIT or IIT) is highly determined on male sexual performance, any limitation imposed on their ability to achieve mating could detrimentally affect IIT perspective. However, the effect of *Wolbachia* on traits related to sexual behavior, such as sexual signaling in medfly, have not been addressed yet.

Despite the numerous studies dealing with the evaluation of either food content or *Wolbachia* infection on the biological traits of several insect species, the interaction between food and *Wolbachia* has not been examined yet. Moreover, considering *Wolbachia* infection as an additional factor that could affect male sexual signaling, and generally the behavioral and fitness traits of the infected insects, *C. capitata* could be a model organism to investigate the interaction between food, infection, and aging in insects. Inasmuch as the effects of *Wolbachia* infection on the medfly sexual behavior and the interaction between *Wolbachia* infection and adult food remain unexplored, we initiated this study to determine the effects of *Wolbachia* on the sexual signaling of protein-fed and protein-deprived males. We compared two laboratory lines with a shared genetic background: (a) “Benakeio”, a *Wolbachia*-uninfected laboratory line, and (b) “WolMed 88.6”, a *Wolbachia*-infected line that was developed from the transinfection of “Benakeio” line with *w*Cer2, a strain of *Wolbachia* originated from *Rhagoletis cerasi* (L.) (donor) [118]. We tested the hypotheses that: (a) *Wolbachia* infection alters (i) the frequency and (ii) the daily pattern of male sexual signaling, and (b) the effects of the *Wolbachia* infection are more pronounced on protein-deprived *C. capitata* males. The answers to these queries could be the basis for future experimentation aiming to elucidate the medfly biological traits under the bacterium influence and the evaluation of IIT perspective, either as a stand-alone or in combination with SIT in pest control strategies.

## 2. Materials and Methods

### 2.1. Experimental Conditions and Ceratitis Capitata Lines

The experiments were conducted in the laboratory of Entomology and Agricultural Zoology at the University of Thessaly, Greece, under standard laboratory conditions, 25 ± 1 °C, 55 ± 5% r.h., and L14:D10 photoperiod, with the photophase starting at 07:00 h. Light was provided by daylight fluorescent tubes with the intensity inside the test cages ranging between 1500 and 2000 lux. We used the following medfly lines: (a) “Benakeio”, a *Wolbachia*-uninfected laboratory strain kept under the same laboratory conditions for more than 30 years, and (b) WolMed 88.6, a *Wolbachia*-transinfected laboratory population carrying the *w*Cer2 *Wolbachia* strain. The infected line was developed from the transinfection of Benakeio line with *w*Cer2. The *w*Cer2 *Wolbachia* strain originated from naturally infected field populations of *R. cerasi* that were used as the donor species for the development and establishment of the infected medfly line WolMed 88.6 [118]. Both medfly lines used in the study share the same genetic background and were kept under the same rearing conditions described by Diamantidis et al. (2008) [52] and Sarakatsanou et al. (2011) [120], as follows. Groups of approximately 100 individuals were kept in wire-screened wooden cages (30 cm × 30 cm × 30 cm) with constant access to water and standard adult diet. Standard adult diet (YS) consisted of yeast hydrolysate, sugar, and water in a 4:1:5 ratio. Egg collection was accomplished using an artificial, red-colored, hemispheric, hollowed, plastic substrate (dome) of 5 cm diameter. The surface of the dome was punctured with 40–50 evenly distributed holes (1 mm Ø). Each dome was fixed on the lid (5.5 cm Ø) of a plastic Petri dish. Water was added inside the Petri dish to maintain humidity levels appropriate for egg survival and female oviposition. A plastic cup containing 0.5 mL of orange juice was added inside the Petri dishes to stimulate female oviposition [124]. Eggs for the experiments were collected by placing the domes inside the rearing cages for 24 h. The eggs were then transferred on a cotton disk that served as bulking agent for larval diet and was placed inside a clean Petri dish. The piece of cotton was previously soaked with larval diet consisted of 200 g sugar, 200 g brewer’s yeast, 100 g soybean flour, 4 g salt mixture, 16 g ascorbic acid, 16 g citric acid, 3 g sodium propionate, and 1 l water [124]. One hundred eggs were placed in each piece of cotton and the Petri dishes were transferred into plastic containers with a layer of sterilized sand, which served as the pupation substrate of the larvae, at the bottom.

### 2.2. Effect of Age and Food Type on Signaling Performance

Soon after emergence, males from each population were transferred into cubic transparent Plexiglass cages (20 cm × 20 cm × 20 cm), with mesh windows at the two sides of the cube for ventilation, and randomly assigned to one of the two food treatments, which were either standard adult diet (YS) or sugar only (S). Flies had *ad-libitum* access to water via a water-soaked sponge. Each test cage (replicate) contained 10 males. The effects of age and food type on the sexual signaling activity were determined by counting the number of males expressing sexual signaling. The determination of the signaling males was based on the extrusion and expansion into a balloon-like structure of the terminal end of the rectal epithelium, as described by Arita and Kaneshiro (1986, 1989) [125,126]. We recorded sexual signaling from 07:00 to 21:00 h daily from adult emergence until day 7, and at days 10 and 15. Each hourly observation included three records with a lag period of approximately 4 min between two successive records. The average of these three counts was used as the datum in subsequent analyses. When an individual died, it was replaced with another of the same age and food treatment. We ran 10 replicates for each strain and each food treatment.

### 2.3. Statistical Analyses

The effect of *Wolbachia* infection (first factor), food (second factor), and age (repeated factor) on the daily average of male sexual signaling was determined by three-way repeated measures analysis of variance (ANOVA) [127]. The effect of *Wolbachia* infection (first factor), food (second factor), and time of day (repeated factor) on the daily pattern of male sexual signaling was also determined by three-way repeated measures ANOVA performed separately on selected days. All relevant assumptions of repeated measures ANOVA have been met. As the results obtained from those analyses were similar, we present data only for adult day 5. All analyses were conducted using SPSS 20.0 (SPSS, Chicago, IL, USA).

## 3. Results

### 3.1. Effect of Infection, Age, and Adult Food

**Figure 1:**
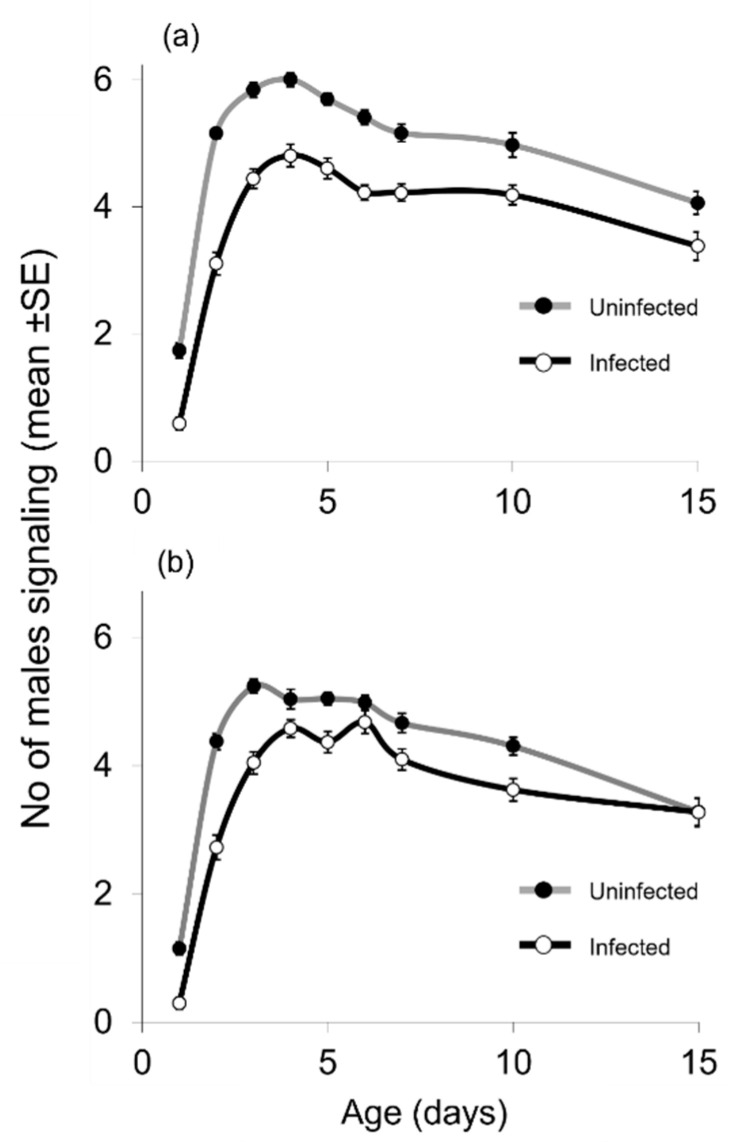
Age-specific sexual signaling of males uninfected and infected with the bacterium *Wolbachia* fed on (**a**) yeast plus sugar and (**b**) sugar only. On each day of age, observations were conducted hourly from 07:00 to 21:00 h in 10 cages (replicates) containing 10 males each. Values on the *y*-axis are mean numbers (±SE) of males signaling per cage per hour observation.

The age-specific signaling rates of *Wolbachia*-infected and non-infected, protein-fed, and protein-deprived males are shown in Figure 1. The first males displaying sexual signaling were recorded already on day 1 of age. In both food regimes and infection status, the frequency of sexual signaling peaked on days 3–6 and then gradually declined up to day 15 (last observation).

Both *Wolbachia* infection and food regime were significant predictors of the frequency of male sexual signaling. The *Wolbachia*-infected males performed less sexual calling compared to the uninfected ones (*F* = 110.8, df = 1, 36, *p* < 0.001). Likewise, the frequency of sexual signaling rates on the protein-deprived males was lower compared to the protein-fed ones (*F* = 24.1, df = 1, 36, *p* < 0.001). The interaction between *Wolbachia* infection and food was also significant (*F* = 6.5, df = 1, 36, *p* < 0.001); however, the partial eta squared value regarding the *Wolbachia* infection alone (partial *η^2^* = 0.755) is much higher than that of the interaction between infection and food (partial *η^2^* = 0.152) or the partial eta squared of food regime alone (partial *η^2^* = 0.401). This indicates that the effect of *Wolbachia* infection is extremely high and probably outweighs any effect of the diet regime. The negative effect of protein deprivation on sexual signaling is actually evident in the uninfected males, whereas the level of sexual signaling is similar in the YS-fed and S-fed infected flies. (Figure 1, Table 1).

Age, used as a repeated factor in the current analysis, was also a sign predictor of the frequency of sexual signaling (*F* = 402.78, df = 8, 288, *p* < 0.001). The interactions between age and infection status (*F* = 10.8, df = 8, 288, *p* < 0.001) and age and food type (*F* = 2.3, df = 8, 288, *p* < 0.024) were both significant (Figure 1, Table 1).

Considering days 1–4 as an indicative period of male sexual maturity, data analysis revealed that both *Wolbachia* infection, food regime, and males’ age significantly affected the sexual signaling rate (*F* = 168.55, df = 1, 36, *p* < 0.001; *F* = 30.80, df = 1, 36, *p* < 0.001; and *F* = 1163.77, df = 3, 108, *p* < 0.001, respectively). The interactions between *Wolbachia* infection and food type (*F* = 4.57, df = 1, 36, *p* = 0.039) and *Wolbachia* infection and age (*F* = 15.90, df = 3, 108, *p* < 0.001) were also significant. In contrast, the interaction between food regime and the age of males was not significant, indicating that the reduced frequency of sexual signaling of the *Wolbachia*-infected males was proportionally similar in both diets during the first four days of their life (*F* = 0.40, df = 3, 108, *p* = 0.753).

### 3.2. Effect of Infection, Food, and Time of Day

**Figure 2:**
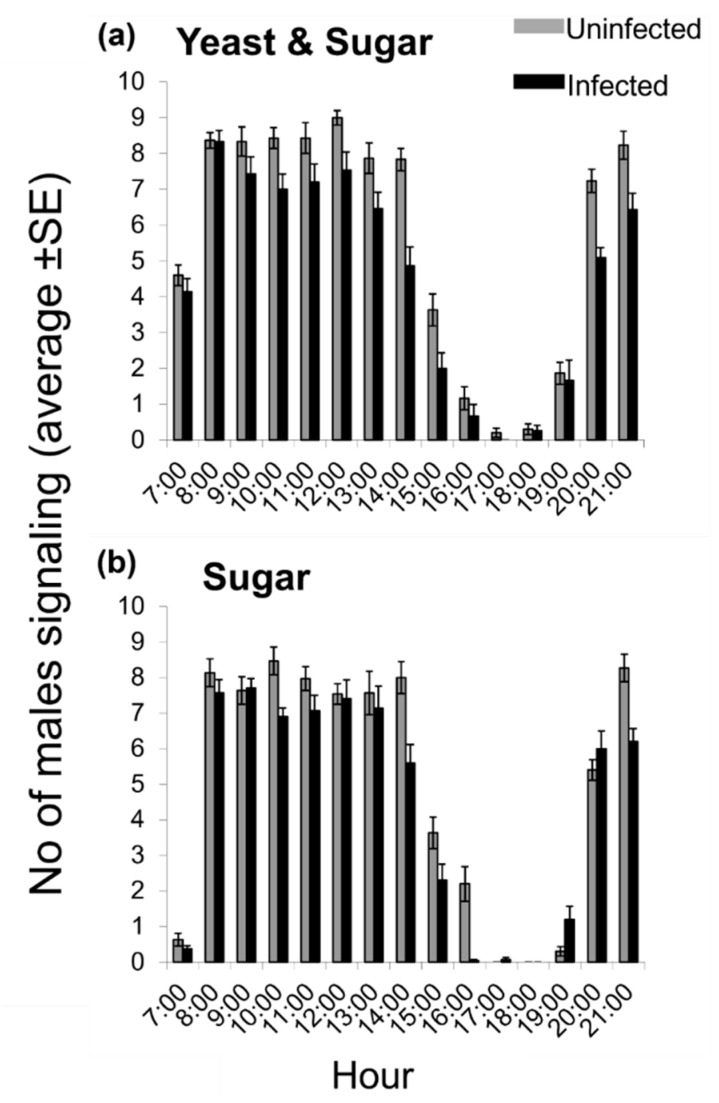
Daily rhythm of sexual signaling on adult day 5 of *Ceratitis capitata* males uninfected and infected with *Wolbachia* fed on a (**a**) yeast plus sugar (YS) and (**b**) sugar only (S) diet. The values on the *y*-axis are mean numbers (±SE) of males signaling per cage per hour observation. The grey and black bars represent the uninfected and infected line, respectively.

The daily patterns of sexual signaling, regardless of adult food and infection status, followed a bimodal pattern, with one broad peak from around 08:00 to 14:00 h and a second one from 20:00 to 21:00 h (Supplementary Materials, Figures S1 and S2). As the daily patterns were similar throughout the experimental days, we analyzed day 5 of the male age as being representative (Figure 2). Considering the records in the most sexually active period (07:00 and 15:00) of day 5, we found that both *Wolbachia* infection and food regime significantly affected the frequency of sexual signaling (F = 31.7, df = 1, 36, *p* < 0.001 and F = 8.5, df = 1, 36, *p* = 0.006, respectively). The time of day was also a significant predictor of male sexual signaling (F = 133.5, df = 8, 288, *p* < 0.001). The significant interaction between *Wolbachia* infection and time of day indicates the impact of *Wolbachia* on the daily rhythm of sexual signaling (F = 3.9, df = 8, 288, *p* < 0.001), whereas the significant interaction between food type and time of day indicates the effect of adult diet on the daily rhythm of sexual signaling (F = 11.6, df = 8, 288, *p* < 0.001). However, the partial eta squared value of time of day (partial *η^2^* = 0.788) is much higher compared to the partial eta squared of the interaction effect between infection and time of day (partial *η^2^* = 0.098). This indicates that the effect of the time of day on the levels of sexual signaling is much stronger that the effect of *Wolbachia* infection. This can be attributed to the fact that the general daily pattern of sexual calling does not change significantly between the infected and the uninfected line in terms of timing during the day, but the levels of sexual signaling for each time point differ, depending on the infection status. Even though the daily patterns of sexual signaling seemed similar for all treatments and strains, regardless of the nutritional or the infection status, there were quantitative differences between the medfly strains and the diets that resulted in the significant differences in our statistical analysis. For instance, YS-fed males started sexual calling at the same time of day with sugar-fed males, but the number of YS-fed calling males was 4–5 times more than the number of S-fed calling males. Similarly, even though sexual signaling started at the same time of day for infected males, signaling rate was much higher in YS-fed infected males than S-fed infected males at 7:00 h. Moreover, uninfected males showed the highest signaling rate at 12:00 h when fed a YS-diet (Figure 2a), but their highest signaling rate was at 10:00 and 21:00 when fed an S-diet (Figure 2b). On the other hand, infected males showed the highest signaling rate at 8:00 when fed a YS-diet (Figure 2a), and at 8:00 and 9:00 when fed an S-diet. The interaction between *Wolbachia* infection and food type was not significant (F = 1.4, df = 1, 36, *p* = 0.245) (Table 2).

## 4. Discussion

Our results demonstrate that: (a) *Wolbachia* infection reduced male sexual signaling rates in both diet regimes; (b) the negative effect of *Wolbachia* infection was slightly more pronounced on protein fed than on protein-deprived males only for day 6, and it was higher at younger ages, indicating that the bacterium may affect male sexual maturity; (c) *Wolbachia* infection alters the daily pattern of sexual signaling; and (d) protein deprivation bears significant descent on the sexual signaling frequency of the uninfected males, whereas no difference was observed for the *Wolbachia*-infected ones. Additionally, even though the interaction between infection status and diet was statistically significant, the effect size of *Wolbachia* alone was much higher, which is also indicated by the inability of a protein-rich diet to counteract the negative effect of *Wolbachia* infection. However, diet can improve sexual calling in uninfected lines, which is why our analysis considered the effect of food and its interaction with *Wolbachia* infection as statistically significant but with a lower partial eta squared value. Similarly, the effect of the interaction between infection and time of day was significant, but the effect size of time of day alone was much higher that the effect size of the interaction between time of day and *Wolbachia* infection or *Wolbachia* infection alone, and this indicates the daily patterns of sexual signaling do not change between the uninfected and the infected males, but the actual levels of sexual calling are reduced in infected males.

In the current study, *Wolbachia* infection induced a significant fitness cost to medfly males by dramatically reducing their sexual signaling rates. A previous study by Kyritsis et al. (2019) [122] showed that the same infected medfly line (WolMed 88.6) demonstrates reduced male mating competitiveness compared to the “Benakeio” uninfected line—which is also the uninfected line that we used for our comparisons. The combination of our results with the study of Kyritsis et al. (2019) [122] are in agreement with findings in other studies that showed that reduced sexual signaling frequency is related to reduced mating success [30,31,32]. On the other hand, the same study showed that the infection of flies of the same genetic background with a different *Wolbachia* strain (*w*Cer4) increased male mating competitiveness. However, our study did not test signaling frequency in this specific medfly line that is infected with *w*Cer4 (known as S10.3); therefore, we cannot proceed with any similar comparison of our findings regarding this strain. Additionally, Kyritsis et al. (2019) showed that (*w*Cer2) *Wolbachia* strain had no significant effect on male sexual performance when introduced in a different medfly genetic background [122]. These contradictory results confirm that the genotype of both the insect host and the *Wolbachia* strain interactively determine mating performance phenotypes.

*Wolbachia* cost in sexual calling was not fixed by protein-rich diets and was highly dependent on the conditions the males were exposed to. Contrary to our initial hypothesis, protein provision did not offer any advantage to the *Wolbachia*-infected males, whereas it increased sexual signaling frequency in uninfected males. Interestingly, the signaling performance of the protein-fed infected males was almost equal to the respective performance of the nutritionally stressed uninfected males. Considering sexual signaling as a determinant component of male sexual success, we assume that sexual signaling can only be decreased down to a specific threshold, which was reached due to one factor (e.g., *Wolbachia* infection), and therefore the presence of more than one stress factor (e.g., *Wolbachia* infection and lack of protein source) may not confer additional disadvantage.

Male medflies with access to protein-rich diets demonstrate enhanced male sexual performance attributes such as high sexual signaling frequencies [50,51,52], increased participation in leks, higher mating success and mating duration, and inhibition of female remating [34,53,54,55,56,57,58]. Kaspi et al. (2000) reported that protein-fed medfly males are more prone to achieve matings than protein-deprived ones because of the increased chances to emit pheromone [35]. The importance of diet quality on sexual signaling rate was also highlighted by Papadopoulos et al. (1998) [51], Diamantidis et al. (2008) [52], and Papanastasiou et al. (2019) [50]. Our findings in uninfected flies agree with these studies that highlighted the positive effect of protein feeding on the frequency of male sexual signaling on different medfly natural populations. On the other hand, both Papadopoulos et al. (1998) [51] and Shelly et al. (2002) [128] showed no positive effect of protein provision on the signaling performance of laboratory-reared males, which is in accordance with our findings for the infected laboratory-reared males but in disagreement with our results for the uninfected laboratory-reared males. In fact, it seems that the nutritional effects on sexual behavior are more likely to be detected when the experiments are conducted with natural than with laboratory-adapted populations [129].

Apart from the effect of *Wolbachia* on the frequency of sexual signaling intensity, our results showed that the bacterium could also modify the daily pattern of the signaling activity. Considering that nutrient acquisition highly determines calling performance [50,51,52], the modification of daily sexual activity could be an indirect effect of *Wolbachia* infection. It has been shown that *Wolbachia* affects host metabolism and nutrient utilization via different ways that range from metabolic parasitism to potential nutritional mutualism [130]. For example, *Wolbachia* is involved in the provision of its host with B vitamins [131], nucleotides [132,133] and heme [134,135], iron homoeostasis of its host [136], and virus protection using alterations on its host cholesterol profile [137]. Moreover, *Wolbachia* competes with its mosquito hosts for amino acids leading to reduced fecundity and egg viability, but its negative impact is counteracted by amino acid supplementation of mosquito diet [138]. Therefore, the readjusted daily signaling pattern in our study could be an associated adaption in order to manage the available nutrients more effectively. In addition, the nutritional role of intestinal bacteria in medfly [139] and the recently confirmed *Wolbachia* effects on the relative abundance of resident bacteria in mosquitoes [140], *Drosophila* [141], and planthoppers [142,143] set the ground to further investigate the interaction between *Wolbachia* and medfly gut microbiota and the impact of this interaction on nutrient utilization and/or metabolism. Moreover, there is evidence that the composition of the symbiotic microflora could affect *C. capitata* mating behavior. More specifically, using mating latency time as an indicator of male mating competitiveness, Ben Ami et al. (2010) showed that *Klebsiella* sp. provision improved male sexual performance [144]. Similarly, Ben Yosef and colleagues (2008) reported that antibiotic-treated males needed longer time to achieve mating compared to the untreated ones [145]. Several studies have also demonstrated the dominance and stability of *Klebsiella* abundance across different wild populations and laboratory strains of medfly [146,147,148] and the presence of genes in *Klebsiella* genome that play a potential role in the acquisition and metabolism of nutrients that are vital for insect fitness and reproductive success [148]. Therefore, *Wolbachia* might have an impact on nutrient utilization either by directly affecting metabolic functions of its host or through the perturbation of the host symbiotic microbiota, and this impact might lead to changes in the sexual behavior and performance of the host. Further investigation of the effect of *Wolbachia* infection on the abundance of other symbionts such as *Klebsiella* might help elucidate the complex impact of *Wolbachia* on medfly sexual performance and overall fitness.

## 5. Conclusions

*Wolbachia*-induced cytoplasmic incompatibility (which is the basis of the Incompatible Insect Technique (IIT)) is currently being considered as a stand-alone tool or in combination with the Sterile Insect Technique (SIT) to suppress populations of insect pests [149,150,151,152,153]. A major prerequisite for the successful application of these methods is the use of sterile males that can compete with wild males for copulations with wild females in the field. Our results indicate that the presence of *Wolbachia* may impose a significant disadvantage on male mating success via the reduction of the sexual signaling rates, the modification of the daily rhythm of sexual calling, and the delay of male sexual maturity. In fact, it has been recently proved by Kyritsis et al. (2019) that some *Wolbachia* strains lead to reduced male mating success, whereas others have no negative effect. It is therefore crucial to thoroughly study all the traits that contribute to male sexual competitiveness by comparing infected and uninfected counterparts before any infected strain could potentially be considered as a tool for an insect pest (or vector) population suppression approach.

## Figures and Tables

**Table 1:** Repeated measures analysis of variance on the effect of infection (first factor), food (second factor), and age (repeated factor) on *Ceratitis capitata* male sexual signaling. Ages from adult day 1 to adult day 15 were considered in the analysis.

Source of Variation	df	MS	F	*p*	Partial Eta Squared
Infection	1	79.0	110.8	<0.001	0.755
Food	1	17.2	24.1	<0.001	0.401
Infection × Food	1	4.6	6.5	<0.001	0.152
Error (between subjects)	36	0.714			
Age	8	67.4	402.8	<0.001	0.918
Age × Infection	8	1.8	10.8	<0.001	0.231
Age × Food	8	0.4	2.3	0.024	0.059
Age × Infection × Food	8	0.2	1.0	0.449	0.027
Error (Age)	288	0.167			

**Table 2:** Repeated measures analysis of variance on the effect of infection (first factor), food (second factor), and hour of the day (repeated factor) on *Ceratitis capitata* male sexual signaling. Times of day from 7:00 to 15:00 (the most sexually active period of the day) of adult day 5 were considered in the analysis.

Source of Variation	df	MS	F	*p*	Partial Eta Squared
Infection	1	100.9	31.7	<0.001	0.469
Food	1	27.0	8.5	0.006	0.191
Infection × Food	1	4.44	1.4	0.245	0.037
Error (between subjects)	36	3.182			
Time of day	8	194.9	133.5	<0.001	0.788
Time of day × Infection	8	5.7	3.9	<0.001	0.098
Time of day × Food	8	16.9	11.6	<0.001	0.243
Time of day × Infection × Food	8	0.8	0.6	0.790	0.016
Error (Time of day)	288	1.460			

## Data Availability

The data presented in this study are available on request from the corresponding author. The data are not publicly available due to the fact that they are part of a Ph.D. thesis.

## Supplementary Materials

The following supporting information can be downloaded at: https://www.mdpi.com/article/10.3390/insects13080737/s1, Figure S1: Daily rhythm of sexual signaling in *Ceratitis capitata* males. Figure S2: Daily rhythm of sexual signaling in *Ceratitis capitata* males.
