# Supplementary material for: Effect of Wolbachia Infection and Adult Food on the Sexual Signaling of Males of the Mediterranean Fruit Fly Ceratitis capitata
Source: Insects. 2022 Aug 17;13(8):737. doi: 10.3390/insects13080737 (PMC9409120; doi:10.3390/insects13080737)
Supplement: Supplementary file 1 [file insects-13-00737-s001.zip › insects-1787410-supplementary.pdf]

Supplementary Material

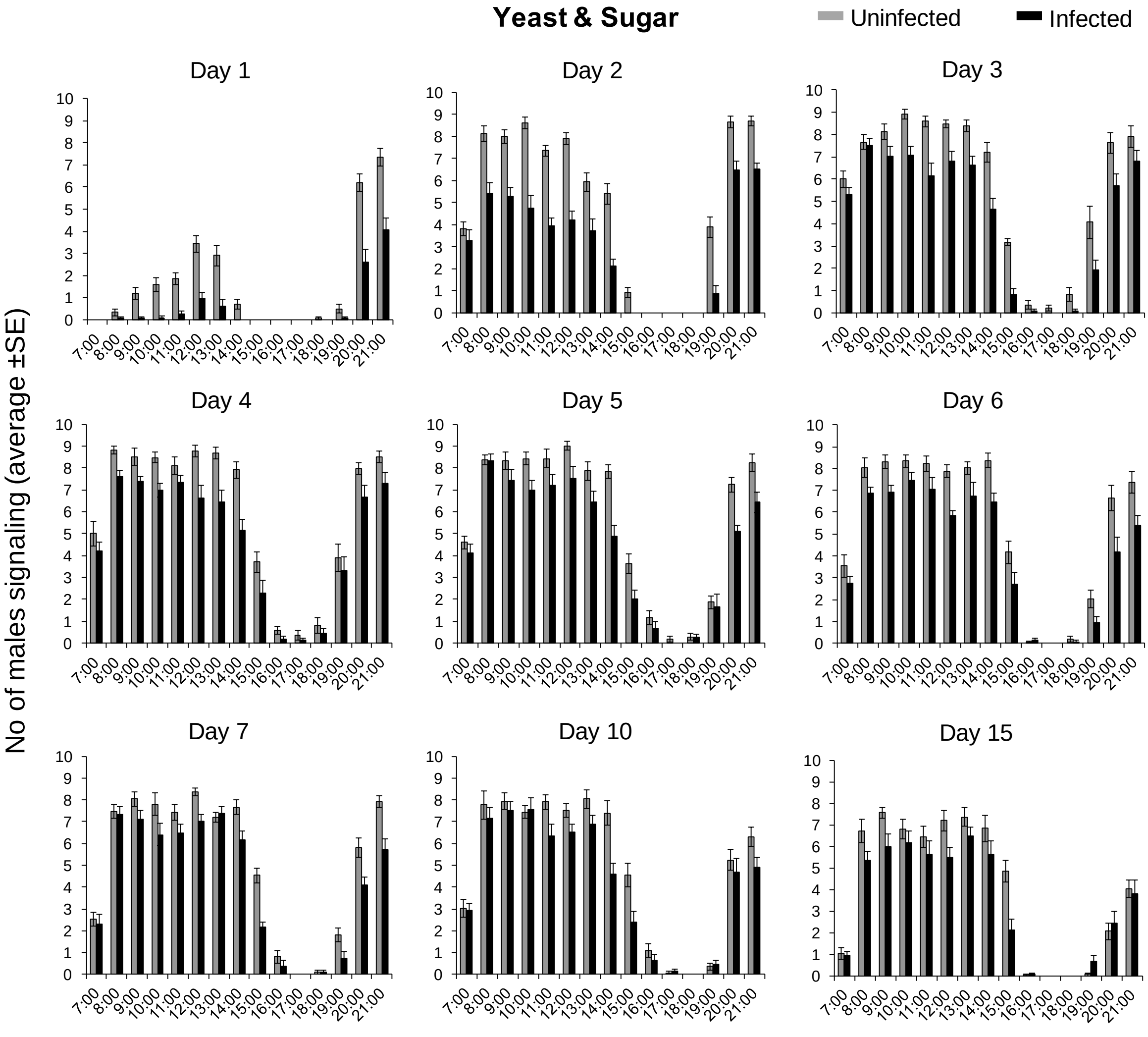

Figure S1. Daily rhythm of sexual signaling in *Ceratitis capitata* males, fed on yeast plus sugar, on days 1-7, 10 and 15 after emergence. Values on y-axis are mean numbers (±SE) of males signaling per cage.

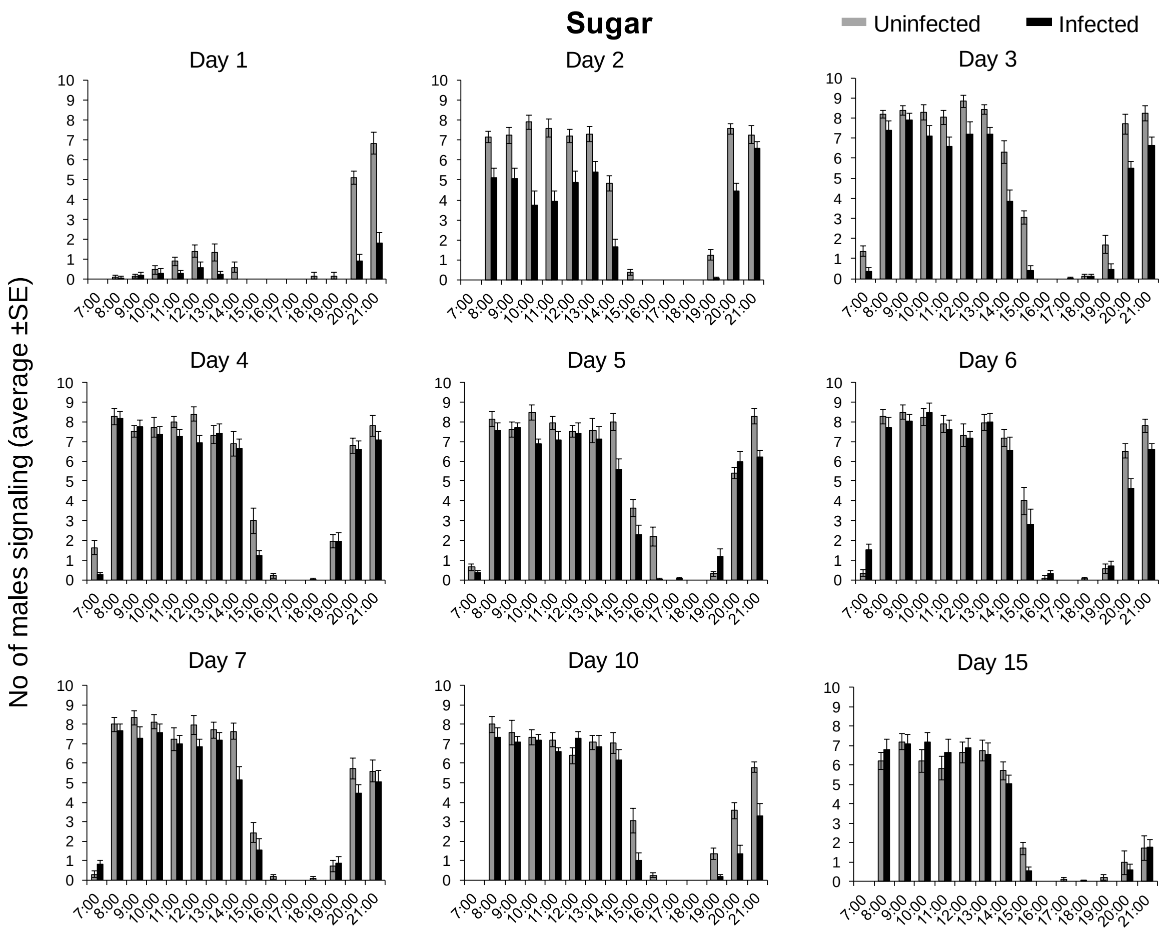

Figure S2. Daily rhythm of sexual signaling in *Ceratitis capitata* males, fed on sugar only, on days 1-7, 10 and 15 after emergence. Values on y-axis are mean numbers (±SE) of males signaling per cage.
